# Balancing under constraint: Structural insights into norovirus evolution and antigenic innovation

**DOI:** 10.1371/journal.ppat.1014383

**Published:** 2026-06-30

**Authors:** Yun Chen, Xin-zi Wang, Ying-yu Tan, Jie-mei Yu

**Affiliations:** College of Life Sciences and Bioengineering, Beijing Jiaotong University, Beijing, China; Medical Research Council Laboratory of Molecular Biology, UNITED KINGDOM OF GREAT BRITAIN AND NORTHERN IRELAND

## Abstract

Norovirus is the leading cause of acute viral gastroenteritis worldwide. While genomic studies have revealed its diversity and evolutionary patterns, the structural mechanisms driving viral adaptation remain poorly understood. Here, we establish a comprehensive structural database of norovirus VP1 P-domains across nine genogroups (GI-GIX) through large-scale AlphaFold2 predictions. By integrating phylogenetic analysis of VP1 sequences and structures, we demonstrate that sequence and structural evolution show overall concordance under purifying selection, yet significant local discrepancies reveal distinct patterns of convergent evolution shaped by structural constraints and functional divergence. Focusing on the predominant GII.4 genotype, we found that compared to near-full-genome and nucleotide trees, only the VP1 amino acid tree reliably clustered GII.4 variants in chronological order as monophyletic groups. We further identify a hierarchical evolutionary strategy: positive selection may drive structural hypervariability in major antigenic epitopes D and C for immune escape, with epitope D exhibiting pronounced structural flexibility that complicates its structural characterization, whereas coevolutionary analysis uncovers a broad network of compensatory interactions spanning multiple epitopes, with striking enrichment in epitope A. These epitopes exhibited a pattern of “sequence plasticity with structural conservation”, maintained by coevolutionary constraints that preserve conformational integrity. Together, these findings suggest that norovirus vaccine strategies targeting the structurally conserved conformations of epitopes A and G could overcome the limitations of traditional strain-specific approaches, offering a pathway toward broad protection against evolving viral diversity.

## Author summary

Noroviruses are a leading cause of acute gastroenteritis worldwide, with the GII.4 genotype persistently dominating outbreaks. How this virus balances the need to escape human immunity while maintaining a functional protein shell (capsid) has remained unclear. Using large‑scale computational predictions of capsid structures, we discovered that GII.4 employs a sophisticated two‑pronged strategy. Key antibody targets (epitopes A and G) maintain a stable three‑dimensional shape, even though their building blocks (amino acids) vary. In contrast, major epitopes D and C exhibit pronounced structural flexibility, which facilitates immune evasion. Remarkably, a broad network of coevolving interactions spans these epitopes, compensating for sequence changes and thereby preserving structural integrity where needed while allowing flexibility elsewhere. Our findings explain why GII.4 has been so successful and suggest that vaccines targeting the structurally stable epitopes could provide broad protection, overcoming the challenge of rapid viral evolution. The approach also illustrates how computational structural biology can unravel the evolutionary strategies of fast‑evading pathogens.

## Introduction

The evolutionary trajectory of viruses is shaped by a fundamental conflict: the imperative to evade host immunity, often through rapid sequence change, is pitted against the stringent structural constraints required to maintain functional integrity [1,2]. Although genomic analyses have richly documented the patterns of viral diversity, the structural principles that resolve this conflict, thereby enabling successful lineages to persist over time, remain a central mystery in evolutionary virology. Noroviruses, with their extensive genetic diversity, defined major antigenic sites, and long-term dominance of the GII.4 genotype [3], present an ideal model system for dissecting these universal principles. Elucidating how noroviruses structurally navigate the selective landscape is crucial not only for combating a major global pathogen but also for uncovering general rules of viral adaptation.

Norovirus is the leading cause of acute viral gastroenteritis worldwide, with approximately 685 million cases annually and posing a substantial burden on public health systems, particularly among children and immunocompromised populations [4,5]. It is a nonenveloped, positive-sense, single-stranded RNA virus belonging to the family *Caliciviridae*. The genome is organized into three or four open reading frames (ORFs) [6,7], of which ORF1 encodes a polyprotein precursor that gives rise to several nonstructural proteins, including the RNA-dependent RNA polymerase (RdRp). ORF2 encodes the major structural protein VP1, which forms the viral capsid and plays a crucial role in interactions with host cells. VP1 contains two functional domains: the shell (S) domain and the protruding (P) domain. The P domain can be further divided into P1 and P2 subdomains, with the P2 subdomain being particularly important as it contains variable antigenic sites and binding sites of histo-blood group antigens (HBGAs) [8,9], making it a critical target for studies on viral evolution and vaccine design.

Based on the VP1 amino acid (AA) sequence diversity, noroviruses are classified into ten confirmed genogroups (GI-GX), of which GI, GII, GIV, GVIII and GIX infect humans, with GII genotypes been the most prevalent. Based on nucleotide diversity in the RdRp, eight confirmed P-groups and at least 60 P-types have been reported to date [10,11]. Recombination is common in noroviruses and typically occurs at the ORF1-ORF2 overlap, giving rise to various strains, such as GII.4[P4], GII.4[P16] and GII.2[P16], which have circulated during periods [12]. Furthermore, point mutations can also induce functional alterations, including prolonged viral shedding and the emergence of immune escape variants [13,14]. Therefore, molecular evolutionary studies based on the norovirus genome are crucial for understanding viral host adaptation, dynamic changes in epidemic strains, and transmission patterns.

Conventional evolutionary analyses primarily rely on viral sequence comparisons. However, excessive sequence divergence and substitution saturation often obscure phylogenetic signals, limiting the resolution of core evolutionary drivers [15,16]. As protein structures evolve more slowly than sequences, structural phylogenetics has emerged as a complementary approach [17]. For noroviruses, advances in structural biology, including X-ray crystallography and cryo-electron microscopy, have enabled the determination of P-domain structures across various noroviruses [18–21], revealing mechanistic insights into receptor binding and antibody neutralization. Nevertheless, due to the inherent high variability and conformational flexibility of the P domain [22], along with the throughput limitations of traditional structural determination methods, comparative analyses of inter- and intra-genotypic structural differences remain challenging, hindering a comprehensive understanding of norovirus structural diversity. AlphaFold has revolutionized protein structure prediction through deep learning algorithms [23,24], providing a powerful platform for large-scale structural database generation. This breakthrough enables systematic prediction of P-domain structures across major norovirus genogroups or epidemic strains. The structural comparison tool Foldseek further facilitates this analysis by converting three-dimensional spatial relationships into quantifiable geometric descriptors [25], allowing efficient evaluation of structural features at scale. This strategy has been successfully demonstrated in flaviviruses, where structural phylogenetics elucidated glycoprotein evolution and revealed cross-genus gene transfer events [26], proving its value for complex viral evolutionary studies.

Given the high genetic diversity of noroviruses, their rapid strain turnover, and the pivotal role of the P-domain (especially P2 subdomain) in host recognition and immune evasion, constructing a comprehensive structural panorama is essential to decipher viral variation patterns and their evolutionary drivers. In this study, we utilize high-quality norovirus sequences from public databases to predict P-domain structures across genotypes and variants using AlphaFold2. We validate model reliability through per-residue predicted Local Distance Difference Test (pLDDT) scoring and perform systematic computational structural comparisons among genotypes and variants to elucidate the core evolutionary forces shaping P-domain diversity. This work aims to uncover intrinsic links among structure, function, and viral adaptability, thereby enhancing our understanding of host adaptation and transmission dynamics. The insights will facilitate the prediction of epidemic trends and provide a scientific foundation for the development of broad-spectrum vaccines and antiviral strategies.

## Results

### Sequence information

To investigate the genetic diversity and evolutionary dynamics of noroviruses, we compiled 4,162 near-complete genome sequences, the vast majority (95.3%) of which were derived from human infections. The remaining sequences originated from animals (e.g., murine, porcine and bovine) and environmental samples. These sequences spanned nine genogroups (GI-GIX), with human-infecting GII being overwhelmingly dominant (88.03%), followed by GI (7.45%) (S1A Fig). Animal-associated genogroups included GIII (bovine), GV (murine), and broadly host-adapted GIV, along with rare GVI and GVII genogroups (from murine and vulpine sources) were also identified, reflecting the broad host range of noroviruses. Temporal analysis of the GI and GII genogroups revealed distinct patterns of genotype dominance across different periods (S1B–S1C Fig). For instance, prior to 2011–2013, GII.4[P4] was the overwhelmingly predominant variant. From 2011-2013 onward, GII.4[P31] emerged and maintained its significant as a major circulating strain. During and after the COVID-19 pandemic, GII.4[P16] and GII.17[P17] became the dominant epidemic variants. Furthermore, we successfully extracted 4,124 high-quality VP1 sequences and 4,121 RdRp sequences from the complete norovirus genomes, which were subsequently used for structural and other related analyses. Sequence analysis revealed that following 100% deduplication, the AA similarity of VP1 ranged from 21.6% to 99.9%, whereas that of the P-domain ranged from 16.8% to 94.4%. This broad range of similarity, particularly the lower end observed for the P-domain, reflects extensive antigenic and functional diversification driven by host immune pressure and provides the foundation for our structural investigations.

### Phylogenetic comparison reveals recombination and topological discordance

A tanglegram based on VP1 and RdRp AA sequences revealed that the VP1 tree clustered all strains according to established genogroup and genotype classifications. However, comparison with the RdRp tree revealed widespread recombination signals. First, inter‑genogroup recombination was observed: although GIX and GVIII are distantly related to GII in the VP1 tree, their RdRp sequences were derived from GII.P. Second, within the predominant GII genogroup, complex recombination patterns were identified at the genotype level. For example, GII.4 VP1 was found to recombine with GII.P4, GII.P31, GII.P16, and GII.P12 polymerases, while GII.17 VP1 recombined with GII.P31 and GII.P17. Recombination events were also detected within the GI genogroup and among other genotypes such as GII.2 and GII.6 (S2 Fig).

**Fig 1:**
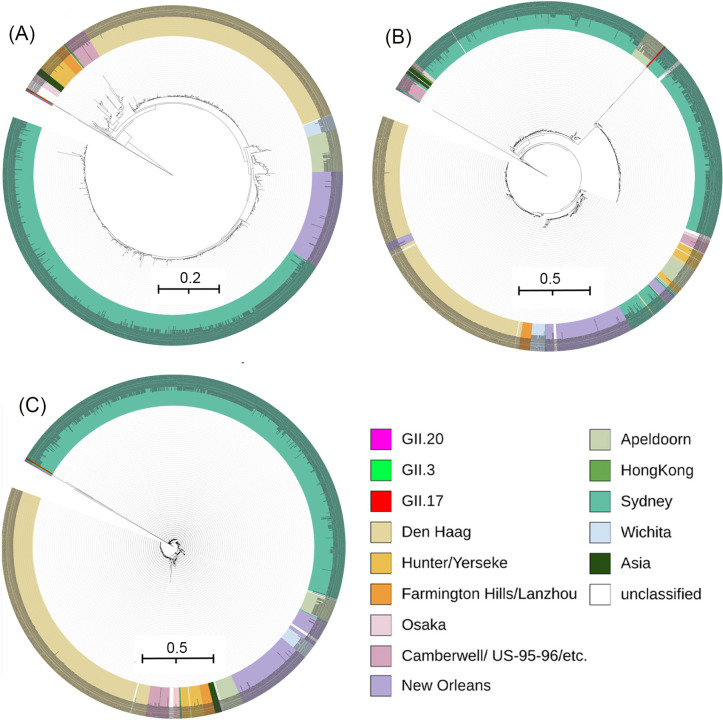
Phylogenetic comparison of GII.4 variants. **(A)** AA tree. **(B)** near-full-genome tree. **(C)** nucleotide tree. In the AA tree, variants form distinct clusters, and their phylogenetic relationships are consistent with their emergence over time. In contrast, the topologies of the full-length and nucleotide trees differ markedly from that of the AA tree. Trees were rooted with GII.17, GII.3, and GII.20 as outgroups.

To further evaluate the impact of recombination on phylogenetic inference, we constructed three trees for the most predominant GII.4 genotype: a near‑full‑genome tree, a VP1 nucleotide tree, and a VP1 AA tree (Fig 1). In the AA tree, the outgroups (GII.17, GII.3, GII.20) were correctly placed, and all variants, such as Sydney, Hunter, Den Haag, and New Orleans, formed distinct monophyletic clusters, and the branching order followed the temporal emergence of variants: early strains (Osaka, Asia) positioned near the root, followed successively by Den Haag, Apeldoorn, New Orleans, and the most recent Sydney. In contrast, the whole‑genome tree exhibited pronounced topological distortions: Apeldoorn was misplaced within the Sydney clade, some New Orleans strains clustered with Den Haag, and even the outgroup GII.17 was nested within Sydney (GII.3 and GII.20 remained correctly placed). The VP1 nucleotide tree showed intermediate discordance: outgroups were properly positioned and most variants formed monophyletic groups, but certain variants (e.g., Wichita) were incorrectly placed within New Orleans. Moreover, neither the whole‑genome tree nor the VP1 nucleotide tree recapitulated the temporal order of variant emergence.

Quantitative analysis of topological differences using normalized Robinson-Foulds (RF) distances further corroborated these observations. The VP1 AA tree showed near‑complete discordance with the whole‑genome tree (RF = 0.859) and high discordance with the VP1 nucleotide tree (RF = 0.840), whereas the VP1 nucleotide and whole‑genome trees exhibited moderate similarity (RF = 0.456). These results indicate that frequent recombination between ORF1 and ORF2 severely distorts the whole‑genome phylogeny.

### The predicted structural models exhibit high confidence

A total of 1,543 tertiary structures of P domains were predicted in this study. The dataset predominantly comprises GII strains (1,272 structures), with GII.4 being the most abundant (659 structures), reflecting both its epidemiological dominance and extensive genetic diversity. Other clinically relevant genotypes, such as GII.3, GII.2, GII.6 and GII.17 were also well represented (131, 110, 109 and 87 structures, respectively), enabling comparative structural analyses across circulating variants. GI contributed 126 structures, while genogroups GIII-GIX collectively accounted for 147 structures, providing a broad evolutionary context. Only GII.26 was excluded due to sequencing ambiguities (non-ATCG nucleotides).

**Fig 2:**
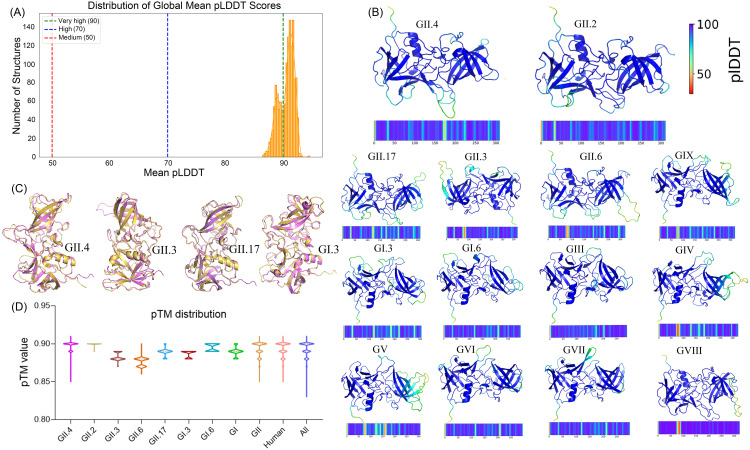
Confidence assessment of the predicted structures. **(A)** Distribution of pLDDT values for all predicted structures. The pLDDT values across all predicted structures had an overall mean of 93.9. **(B)** Residue-level pLDDT values for the major genotypes/groups. Most regions exhibited high confidence, with only a few loop regions showing lower values. **(C)** Structural comparison between predicted models of major genotypes and experimentally determined structures. The overall structures are highly consistent, though some local deviations were observed in loop regions. The experimentally determined structures were retrieved from the Protein Data Bank (https://www.rcsb.org) with accession numbers 5IYR (GII.4), 7ER0 (GI.3), 6IR5 (GII.3), and 5F4J (GII.17). **(D)** Distribution of predicted TM-scores (pTM). All predicted structures exhibited pTM above 0.85.

Per-residue pLDDT scores were used to evaluate the confidence of the predicted structures. Across all models, mean pLDDT values ranged from 86.4 to 98.1 (global mean 93.9), indicating consistently high prediction reliability (Fig 2A). Residue-level heatmaps revealed that high confidence extended across most of the P-domain, with the exception of the first five to six N-terminal residues, a known AlphaFold artifact (Fig 2B), and a few loop regions where pLDDT occasionally fell below 70 (Table 1). Importantly, these low-confidence loops correspond to structurally flexible regions, which are inherently difficult to model but do not compromise the overall fold. Structural alignments between predicted models and experimentally determined counterparts showed close agreement across core secondary structure elements, with minor deviations confined to loops (Fig 2C). Furthermore, the predicted template modeling score (pTM) exceeded 0.85 for all structures (Fig 2D), indicating that the global topologies are highly consistent with native conformations. Together, these validations establish a robust structural foundation for subsequent evolutionary and antigenic analyses.

**Table 1:** Predicted low-confidence regions (pLDDT < 70) across norovirus genogroups/genotypes.

Genogroups /genotypes	Residues with pLDDT <70	Secondary structure
GII.4	171-177 (map to epitope D)	Loop
GII.2	33-36	Loop
GII.17	33-37, 119-127, 156-157, 174-176, 222-224	Loop
GII.3	33-36, 77-86, 182-184, 232, 290-293, 298	Loop
GII.6	35, 75-88, 188-190, 233-237, 293-297	Loop
GI.3	34-37, 117-122, 184-190, 217-220, 277-281	Loop
GI.6	73, 144, 178-182, 271-274	Loop
GIII	175-176, 203-204	Loop
GIV	76-86, 130-132, 163-164, 296-298	Loop
GV	72-82, 121-127, 139-143, 156-167, 189	Loop
GVI	76-99, 100-108, 154-155, 190-191, 226-228	Loop
GVII	36, 120-121, 212-214	Loop
GVIII	73-90, 236-240	Loop
GIX	36, 75-84, 131-132, 233-234	Loop

### Sequence and structure phylogenies support norovirus classification but reveal discrepancies in specific phylogenetic relationships

**Fig 3:**
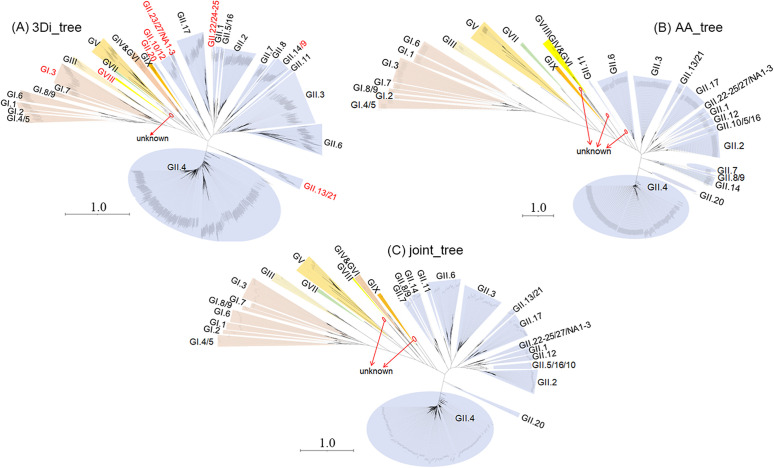
Structurally aligned P domain phylogenies. (**A**) 3Di_tree; **(B)** AA_tree; **(C)** AA + 3Di joint_tree. All three trees are consistent with the established viral classification but reveal discrepancies in specific phylogenetic relationships. Genotypes exhibiting inconsistent placements across the trees are highlighted in red. Sequences that could not be classified are labeled as “unknown” in each tree.

Based on P-domain structural data, we constructed three phylogenetic trees: a 3Di structure-based tree, an AA-based tree, and a combined AA + 3Di tree. All three trees consistently clustered the nine genogroups (GI-GIX) into distinct monophyletic clades without cross-genogroup mixing (Fig 3A–3C). Comparison with the full-length VP1 AA tree revealed overall consistency at the genogroup and genotype levels: for example, GII.4 and GII.20 were closely related in both trees, whereas GII.3 and GII.6 were more distantly positioned. However, minor local discrepancies were observed: GII.14, which was close to GII.4 in the VP1 tree, appeared more distant in the P-domain tree (S3 Fig). The local differences are suggestive of the heightened immune selection pressure acting on the P-domain. GI and GII from human were the most genetically distant genogroups in both AA and 3Di trees, consistent with their early evolutionary separation, while GI and GIII (primarily bovine) maintained relatively close distances across both trees, suggesting a shared ancestral host range or conserved structural constraints. However, notable discrepancies between sequence-based and structure-based topologies were observed: the closest relative to GII differed markedly between trees, with GIX in the 3Di tree versus GIV/GVI in the AA tree. Additionally, GVIII clustered with GIV and GVI in the AA tree but showed greater distance in the 3Di tree. This discordance points to convergent structural evolution, where distinct genetic lineages have arrived at similar three‑dimensional conformations under shared functional pressures.

Within genogroups, most major genotypes exhibited congruent phylogenetic positions across all trees, but several rare genotypes displayed striking discrepancies. For instance, GII.22-25/27 and GII.NA1-NA3 formed tight clusters in the AA tree but split into distantly related clades in the 3Di tree, implying that despite close genetic relatedness, their P domains have undergone substantial structural divergence. Similarly, GII.10 clustered with GII.5/16 and GII.12 in the AA tree but remained proximate only to GII.12 in the 3Di tree, suggesting that structural similarity may be retained even when sequence relatedness fades, a hallmark of functional convergence. The most compelling example involved GII.4, the dominant epidemic genotype. In the AA tree, GII.20 appeared as its closest relative; however, in the 3Di tree, GII.13 and GII.21 emerged as the structurally closest neighbors despite being genetically more distant. This sequence-structure decoupling strongly suggests that immune‑driven selection on the P‑domain has led to convergent structural solutions in independently evolving lineages, enabling similar antigenic surfaces to emerge from different genetic backgrounds.

### Structural divergence across norovirus genogroups and genotypes

**Fig 4:**
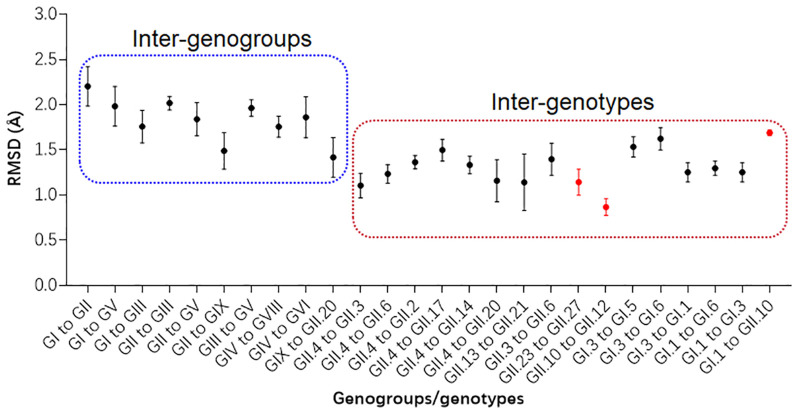
Mean RMSD among genogroups and major genotypes. The structural divergence between different genogroups is overall greater than that observed between different genotypes within the same genogroup. The highest inter-genogroup mean RMSD was observed between GI and GII, while the lowest was between GII and GIX. Among the major circulating genotypes, the smallest mean RMSD was found between GII.4 and GII.3, and the largest between GII.4 and GII.17.

Comprehensive analysis of global mean RMSD values demonstrated that structural divergence between genogroups generally exceed that observed between genotypes (Fig 4). For instance, RMSD for GII versus GIII, GII versus GI, and GIV versus GVIII were consistently larger than those between genotypes within the same genogroup, indicating that genogroup-level classification reflects deep evolutionary divergence in capsid architecture. The closest inter-genogroup relationship was observed between GII and GIX, with a mean RMSD of 1.487 Å, which was lower than certain intra-genogroup comparisons such as GII.4 versus GII.17 and GI.3 versus GI.5 (1.497 Å and 1.532 Å, respectively). This structural similarity between GII and GIX may explain their shared ability to infect humans and suggests conserved functional constraints across these genogroups. The greatest structural divergence was also observed between GI and GII genogroups, with a mean RMSD of 2.203 Å.

Global structural similarity generally correlated with the established phylogenetic relationships. Genogroups that clustered closely in both AA and 3Di trees typically exhibited lower RMSD values. For example, GI and GIII showed an RMSD of 1.757 Å, remarkably smaller than that between GI and GV (1.982 Å). Similarly, the RMSD between GII and GV (1.839 Å) was lower than that between GII and GIII (2.017 Å). Interestingly, despite clustering closely in both AA and 3Di trees, GIV and GVI demonstrated a relatively high RMSD of 1.896 Å, which exceeded values observed for more evolutionarily distant genogroup comparisons such as GIV to GVIII (mean RMSD = 1.756 Å) and GI versus GIII (mean RMSD = 1.757 Å). This discordance suggests that structural divergence can occur despite sequence similarity, potentially driven by adaptation to different host environments, as GIV infects both humans and animals while GVI is animal-specific.

Among major GII genotypes, GII.4 and GII.3 showed the highest structural similarity (mean RMSD = 1.104 Å), while GII.4 and GII.17 exhibited greater structural divergence (mean RMSD = 1.497 Å). Although GII.13 and GII.21 formed tight clusters in both AA and 3Di trees, their mean RMSD values and associated standard deviations were relatively large, indicating a higher degree of structural differentiation. This structural heterogeneity among closely related genotypes may underlie differences in receptor binding or immune escape potential. Within GI genogroup, comparisons of GI.3 with GI.6 and with GI.5 displayed relatively great structural divergence (Fig 4).

**Fig 5:**
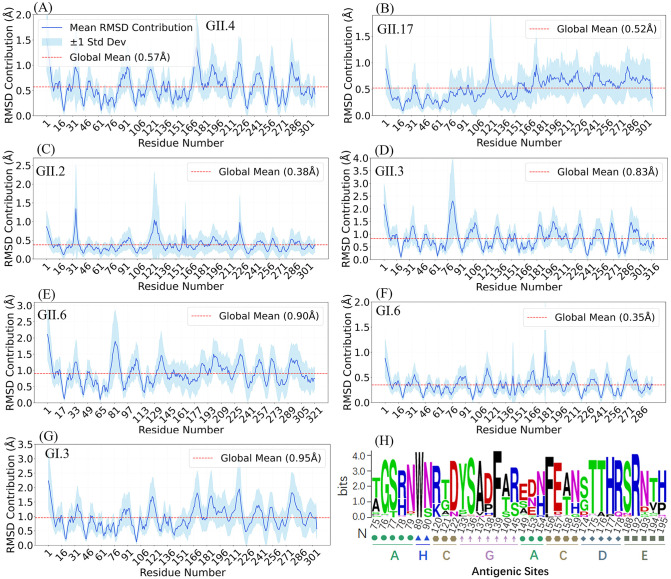
Residue conservation analysis. **(A-G)** The per-residue RMSD contribution. Genotype GII.4, GII.17, GII.2, GII.3; GII.6, GI.6 and GI.3. GII.2 and GI.6 exhibited the highest structural conservation, followed by GII.17 and GII.4. **(H)** Informative AA substitutions. Residues mapping to the major antigenic sites of GII.4 showed substantial diversity.

To further elucidate hypervariable and conserved regions within the norovirus P domain, we analyzed per-residue RMSD contributions from structures of major GI and GII genotypes. The results revealed substantial differences in structural conservation among GII genotypes. GII.2 exhibited the highest structural conservation, with a mean RMSD of 0.38 Å, followed by GII.17 and GII.4, which showed moderate conservation with mean RMSD values of 0.52 Å and 0.57 Å, respectively. In contrast, GII.3 and GII.6 demonstrated considerably higher structural diversity among their variants, with mean RMSD values reaching 0.83 Å and 0.90 Å, respectively (Fig 5A–5E). Within the GI genogroup, we observed a similar pattern of varying structural conservation. GI.3 displayed relatively high structural divergence with a mean RMSD of 0.95 Å, whereas GI.6 showed remarkable structural conservation, with a mean RMSD of only 0.35 Å between variants (Fig 5F–5G). These contrasting structural dynamics suggest that norovirus genotypes employ distinct evolutionary strategies, with implications for their antigenic variability and epidemic potential.

### Structural and selective pressure analysis reveals distinct patterns in GII.4 major antigenic sites

**Fig 6:**
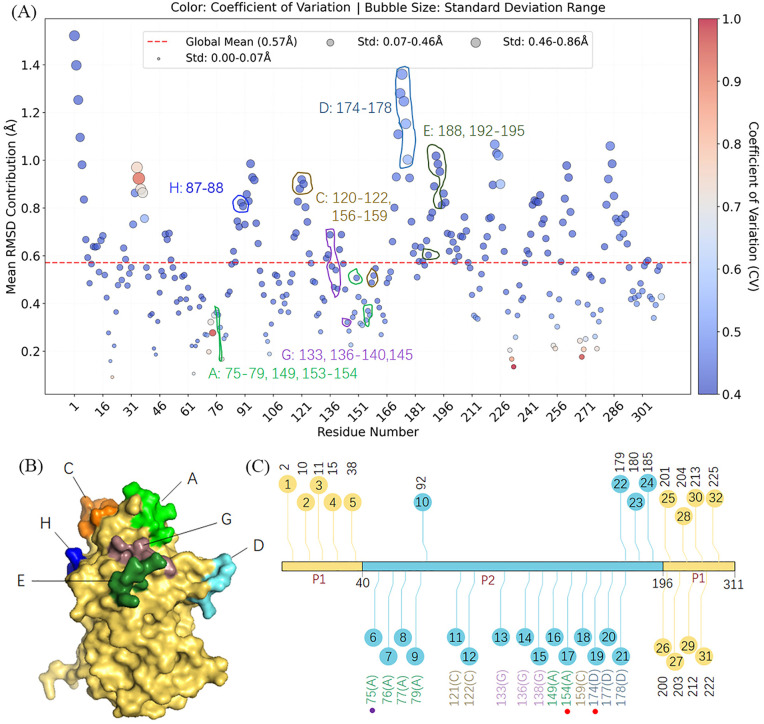
Structural and selective pressure analysis of the P region of GII.4. (A) residue conservation mapping. Localized regions of high structural variability generally corresponded to known antigenic epitopes. **(B)** Spatial distribution of the major antigenic sites. **(C)** Distribution of positively selected sites. Among the 32 positively selected sites identified in the P domain, 19 are located in the P2 subdomain, with 15 falling within the major antigenic epitopes. “Purple circle” marked sites were identified by two methods, and “red circle” marked sites were identified by all three methods, and the rest unmarked sites were identified by only MEME method.

The GII.4 P2 subdomain contains five major antigenic epitopes A, D, C, E and G (Fig 6B). Residue-level RMSD contribution analysis revealed that localized regions of high structural variability generally corresponded to known antigenic epitopes. The most variable region (residues 173–178) mapped to epitope D and exhibited high RMSD standard deviation. Additional peaks of high RMSD values aligned with epitopes H, C, and E, either completely covering these epitopes or encompassing adjacent regions (Fig 6A). Interestingly, despite showing substantial AA diversity (Fig 6H), epitopes A and G demonstrated remarkable structural conservation across GII.4 variants, particularly evident for epitope A (Fig 6A).

Selection pressure analysis identified 32 positively selected sites in the P-domain, 15 of which fell within major antigenic epitopes: 6 in epitope A, 3 in epitope C, 3 in epitope G, and 3 in epitope D (Fig 6C). Of note, the structural impact of positive selection differed markedly between the two classes. Positively selected sites in epitope D (e.g., 174, 177, 178) were located in regions with elevated RMSD (>0.9 Å), whereas those in epitopes A and G (e.g., 75–77, 79, 133, 138, 149) exhibited low RMSD (<0.47 Å) (Fig 6A).

The structurally hypervariable region corresponding to epitope D is located in a region of low prediction confidence (pLDDT < 70). To assess whether the high RMSD values observed in this region reflect genuine biological variation or prediction uncertainty, we compared the structural variability of representative GII.4 variants (including two New Orleans, one Den Haag, and two Sydney strains) using their highest‑confidence models (S4A Fig), as well as the conformational differences among five AlphaFold‑predicted models generated for the same Sydney sequence (S4B Fig). The results showed that for the epitope D region, the average RMSD among different models of the same sequence reached 4.69 ± 2.86 Å, whereas the average RMSD for a control region with high pLDDT (residues 300–307) was only 0.64 ± 0.21 Å. Meanwhile, the average RMSD for this region across different variants was 2.21 ± 1.26 Å. These findings indicate that the high RMSD variability observed in epitope D likely arises from intrinsic uncertainty in modeling this flexible loop, rather than reflecting stable structural differences among epidemic variants.

### Compensatory coevolution within the major antigenic epitopes

**Fig 7:**
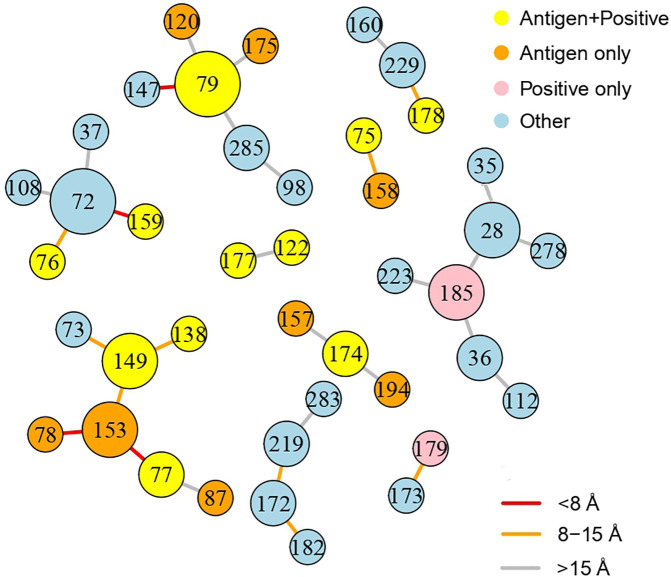
Coevolving residue pairs and potential structural interactions in GII.4 P domain. Among 31 coevolving pairs identified, 17 involve antigenic sites, predominantly in epitope A (12 pairs). Four pairs are in close spatial proximity (<8 Å), suggesting direct structural compensation. Four of the seven hub sites located in the major epitopes.

To investigate whether the major antigenic epitopes are shaped by covarying selection, we performed coevolution analysis on the GII.4 VP1. A total of 51 coevolving pairs were identified, 31 of which lie within the P‑domain. Among these, 17 pairs involve antigenic sites, with a striking enrichment in epitope A (12 pairs), whereas epitopes C and D each contributed 5 pairs and epitope G contribute 1 pair (S1 Table). We further identified seven hub sites with multiple connections: residues 28, 72, 79, 149, 153, 174 and 185, of which three sites (79, 149, 153) are located in epitope A and one site (174) in epitope D. Examination of Cα distances showed that four pairs are in close spatial proximity (<8 Å), 77–153 (5.5 Å), 78–153 (4.8 Å), 79–147 (6.7 Å) and 72–159 (6.3 Å), suggesting direct structural compensation through physical interactions such as hydrogen bonds or hydrophobic contacts. Other pairs are more distant and may be linked via allosteric effects or shared functional interfaces (Fig 7).

## Discussion

The P domain of norovirus VP1 serves as the critical interface for virus-host interactions, playing essential roles in receptor binding and immune recognition [27,28]. This region exhibits substantial genetic variability that underlies antigenic drift [29,30]. Although previous phylogenetic investigations have delineated norovirus diversity and evolutionary patterns at the genomic level [31,32], the structural mechanisms underlying viral persistence, particularly the sustained dominance of the GII.4 genotype, have remained poorly understood. Therefore, we integrated large-scale AlphaFold2 structure prediction, computational structural alignment, and phylogenetic analysis to systematically characterize the genetic diversity and structural evolution of the P domain, revealing complex interrelationships between sequence variation and structural conservation.

We established a comprehensive structural database covering P-domains across nine norovirus genogroups (GI-GIX) using AlphaFold2. Model evaluation confirmed high prediction confidence, with mean pLDDT scores of 93.9 and pTM values exceeding 0.85, indicating reliable global fold topology and atomic-level accuracy, particularly in backbone conformations, comparable to experimentally determined structures [33]. Certain loop regions exhibited pLDDT values below 70, indicating localized conformational uncertainty. Comparative analysis with experimentally solved structures verified high consistency in core domains, with discrepancies primarily confined to these flexible loop regions [34]. The high-quality structural database provides a robust foundation for comparative structural analysis.

Noroviruses undergo frequent recombination at the ORF1-ORF2 junction [35]. In this study, the tanglegram based on VP1 and RdRp also revealed widespread recombination events across different genogroups, particularly within the GII genogroup. The presence of recombination poses challenges for the evolutionary analysis of noroviruses. Some studies have pointed out that the current dual-typing system may not fully capture the full spectrum of recombination events and that whole-genome phylogenetic analysis could improve the accuracy of evolutionary inference [36]. However, by comparing the topological structures of the whole-genome tree, the VP1 nucleotide tree, and the VP1 AA tree for the predominant GII.4 variants, we found that the whole-genome tree failed to cluster major variants into monophyletic groups or recapitulate their temporal order of emergence, likely reflecting conflicting evolutionary signals introduced by recombination between ORF1 and ORF2. The VP1 nucleotide tree retained partial clustering information but, due to the accumulation of synonymous mutations, could not accurately reflect protein-level evolutionary relationships. Only the VP1 AA tree clearly resolved each variant into monophyletic groups following their chronological emergence. This finding is consistent with a large-scale study of GII.4 strains in the United States, which also demonstrated that VP1 AA sequences effectively distinguish different variants and cluster them in temporal order [37]. Notably, the Nextstrain platform explicitly states in its official documentation that whole-genome trees for highly recombinant viruses such as norovirus should be interpreted with caution and suggests combining specific gene-based analyses for more reliable insights [38]. Therefore, while whole-genome sequences contain richer information, recombination signals across different genomic segments may interfere with accurate evolutionary inference. In noroviruses, phylogenetic reconstruction using the functionally constrained VP1 AA provides a more robust evolutionary framework, serving as a valuable complement to whole-genome approaches and representing an effective strategy for focusing on functional regions in evolutionary studies of recombinant viruses.

Our constructed AA, 3Di, and joint trees demonstrated highly consistent topologies across genogroups. This cross-hierarchy consistency indicates that purifying selection maintaining the fundamental protein fold architecture represents the dominant evolutionary force [39, 40]. Nevertheless, beyond this overall consistency, localized discrepancies in specific genogroup and genotype relationships revealed deeper evolutionary dynamics. For instance, GVIII clusters with GIV and GVI in AA trees but appears more distant in 3Di trees, potentially reflecting either rapid structural divergence during environmental adaptation or technical artifacts due to low confidence scores in 73–90 region of GVIII. A particularly striking observation involves the phylogenetic relationships of GII.4: GII.20 appears as its closest relative in the AA tree but is distantly related in the 3Di tree, whereas the genetically more distant GII.13/GII.21 emerges as structurally most similar to GII.4. This sequence-structure decoupling strongly suggests convergent evolution, whereby distinct genotypes independently evolve similar three-dimensional conformations through different AA substitution pathways to adapt to comparable functional pressures. Similar convergent evolutionary patterns have been extensively documented in SARS-CoV-2 Omicron variants and influenza viruses [41–43]. Additionally, certain rare genotypes (e.g., GII.22-25/27) form tight clusters in the AA tree but separate into distinct, distantly related groups in the structural tree, potentially indicating functional divergence driving structural specialization beyond sequence-based predictions.

RMSD analysis serves as a fundamental statistical metric for comparing protein conformations [44,45]. Large-scale computational structural comparisons systematically delineated the landscape of structural variation across norovirus genogroups and genotypes in this study. While recent experimental studies reported Cα RMSD values ranging from 0.586 Å (GII.23-GII.27) to 1.645 Å (GII.9-GX) among five P domains [18], our computational results established that mean RMSD values between major genogroups/genotypes span 0.773-2.419 Å. The general pattern revealed greater structural divergence between genogroups than between genotypes, reflecting overall concordance between sequence and structural evolution. The exceptional case of GII-GIX comparison exhibiting lower RMSD than some intra-genogroup comparisons suggests these genogroups may share a relatively recent common ancestor. This finding is consistent with phylogenetic analyses from both this study and previous studies [36,11], indicating maintained structural similarity that may underlie their shared capacity for human infection.

Intriguingly, experimental structural data reported RMSD values of 1.92 Å for GI.1-GI.10 and 0.4 Å for GII.10-GII.12 [46], while another study documented 0.64 Å for GII.10-GII.12 [47]. Our calculated mean RMSD values for the two pairs (1.759 Å and 0.866 Å, respectively) complement rather than contradict these experimental measurements. This discrepancy highlights the distinction between computational predictions capturing statistical distributions of structural differences across genotypes and experimental data representing specific instances within this distribution. The strong agreement between our GI.1-GI.10 RMSD (1.759 Å) and experimental values (1.92 Å) validates our predictive approach, whereas the lower experimental values for GII.10-GII.12 (0.4-0.64 Å) indicate the existence of specific variant pairs with exceptional structural conservation in nature. This demonstrates the advantage of large-scale computational analysis in revealing population-level evolutionary trends that transcend limitations of individual sample comparisons. Furthermore, elevated RMSD between GIV and GVI despite their close phylogenetic clustering suggests structural divergence following shared ancestry, potentially driven by adaptation to different host selection pressures, GIV infecting both humans and animals while GVI infects animals [48–50]. Similarly, substantial structural differences between genetically similar GII.13 and GII.21 indicate localized adaptive evolution shaping structural outcomes beyond sequence-based predictions.

Antigenic variation in the predominant GII.4 genotype primarily localized to the major epitopes (A, C, D, E, G) within the P2 domain [9,30]. Functionally, epitopes A and G serve as immunodominant sites, whereas epitopes D and E play a regulatory role in antigenicity and may underlie differences in receptor binding potential [9,51,52]. Our study reveals that despite frequent AA substitutions and widespread positive selection across these epitopes, their evolutionary strategy transcends simple antigenic drift, instead exhibiting a sophisticated structural balancing mechanism. The immunodominant epitopes A and G maintain remarkable structural conservation despite bearing the strongest positive selection pressure; epitope A contains the highest density of positively selected sites, including residue 154 robustly identified by all three detection methods. Although epitopes D and C demonstrate classical immune escape patterns with positive selection directly driving local structural variability, it is worth noting that epitope D is located in a flexible loop region with low pLDDT values. By comparing multiple AlphaFold models of the same sequence, we found that structural variation in this region was even greater than the differences among the highest-confidence models of different variants. Therefore, the structural variability observed in this region likely arises from inherent uncertainty in modeling flexible regions rather than reflecting stable structural differences among epidemic variants.

To determine whether structurally conserved epitopes are under compensatory or covarying selection, we further performed coevolutionary analysis on GII.4 VP1, focusing on covarying relationships among major antigenic epitopes. The results revealed that compensatory interactions operate across the entire antigenic landscape. The striking enrichment of co-evolving pairs within epitope A indicates that immunodominant, structurally constrained sites undergo coordinated sequence variation, allowing antigenic diversification while maintaining conformational integrity, a mechanism we term “compensatory fine-tuning” within the “sequence plasticity with structural conservation” paradigm. Concurrently, five coevolving pairs were detected within the structurally variable epitope C, suggesting that it may evade antibody recognition through coordinated structural remodeling. Five coevolving pairs were also identified in epitope D; however, given that this region lies within a flexible loop of low prediction confidence, its coevolutionary signal more likely reflects overall conformational plasticity rather than fine compensatory mechanisms among specific residues. Interestingly, network analysis identified seven residues as evolutionary hubs, of which four are located within major antigenic epitopes, and those within structurally conserved epitopes (79, 149, 153) may serve as “structural anchors” that coordinate compensatory mutations to preserve local conformation [53], whereas hub 174 in the hypervariable epitope D, despite the difficulty of precisely mapping its interactions due to regional flexibility, may still function as a “remodeling center” involved in regulating antigenic variation. Remarkably, both hubs 79 and 149 also exhibited strong positive selection signals and were involved in both short-range and long-range coevolving pairs, suggesting they integrate local structural compensation with global allosteric communication. These observations imply that a small number of key residues act as evolutionarily privileged sites, integrating signals across conserved and variable regions to balance immune evasion with structural integrity. This framework coherently explains why monoclonal antibodies targeting epitope A retain blocking activity against all GII.4 variants [54] and provides structural insights into the hierarchical antibody immunodominance shifts observed across different GII.4 epidemic periods [13].

The above structural insights of GII.4 suggest promising vaccine design strategies. The conserved conformational architecture of epitopes A and G presents an ideal target for developing broad-spectrum vaccines that could elicit cross-protective immunity against both current and emerging GII.4 variants. Furthermore, targeting the stable structural core common to multiple epitopes may help overcome the limitations of traditional strain-specific approaches and provide more durable protection against norovirus evolution.

In summary, our integrated analysis combining large-scale AlphaFold2 predictions with computational structural comparisons reveals structural constraint-adaptation balance as the central driving force in norovirus evolution, specifically elucidating how GII.4 maintains transmission advantage through a hierarchical strategy conserving critical epitope structures while permitting antigenic loop variability. We note that our analysis based on monomeric static P-domain models cannot capture potential effects of dimeric interfaces and conformational dynamics. Future investigations employing full capsid or dimeric modeling, molecular dynamics simulations, and experimental validation of key antigenic epitopes will provide more comprehensive functional insights and establish a solid theoretical foundation for designing broad-spectrum vaccines targeting conserved structural epitopes. The computational framework established here can be extended to other viral systems, offering new paradigms for understanding pathogen adaptive evolution.

## Methods

### Sequence set curation

To construct a comprehensive dataset of norovirus, we retrieved all complete or near-complete genome sequences (≥7000 nt) from the NCBI GenBank Database across the whole history from 1968-2025 (http://www.ncbi.nlm.nih.gov/genbank) using the keyword “norovirus”. The full VP1 and full RdRp regions were respectively trimmed from the raw complete sequences. Sequences containing terms such as “clone”, “patent”, “synthetic”, “plasmid”, “artificial” or “engineered” that indicated manually modified strains were excluded. Further quality filtering removed sequences with ambiguous bases (Ns), gaps, or frameshift-inducing insertions and/or deletions. All retrieved sequences were annotated with its accession number, collection dates, and geographic locations, followed by multiple sequence alignment using MAFFT (http://mafft.cbrc.jp/alignment/server, v7). The P-domain regions were extracted from the VP1 AA sequences, and redundant sequences with 100% identity were removed using CD-HIT.

### Phylogenetic analysis

To investigate recombination events of norovirus, we first reduced redundancy in the VP1 and RdRp AA datasets using CD-HIT with a 98% sequence identity threshold. Phylogenetic trees were then reconstructed separately using IQ-TREE, with the best-fit substitution model automatically selected by ModelFinder, and branch support was assessed with 1,000 ultrafast bootstrap replicates. Both trees were rooted using three sapovirus sequences as outgroups. A tanglegram was subsequently generated to compare the topologies of the VP1 and RdRp trees and to visualize potential recombination events.

To evaluate the evolutionary relationships among GII.4 variants and the impact of recombination on phylogenetic signals, we constructed three phylogenetic trees: a near‑full‑genome tree, a VP1 nucleotide tree, and a VP1 AA tree. Sequences were aligned after redundancy removal at a 100% identity threshold. All trees were inferred with IQ‑TREE, and for consistent comparison, all three trees were rooted using GII.17, GII.3, and GII.20 as outgroups. To quantitatively assess topological congruence, we converted each tree to an unrooted representation using the *ape* package in R and calculated normalized RF distances, which range from 0 (identical topologies) to 1 (completely different topologies). All trees were visualized using iTOL (https://itol.embl.de).

### Structure prediction

The non-redundant sequences were submitted for structural prediction via the AlphaFold2 batch prediction Colab notebook (AlphaFold2_batch.ipynb - Colab). Structural predictions were performed for each sequence utilizing the ColabFold (v1.5.5) package, an implementation of AlphaFold2 (v2.3) [55], two models per target were generated using default parameters, and computations were performed on Google Colab cloud resources. pLDDT is a per-residue confidence score ranging from 0 to 100, reflecting the model’s prediction accuracy for the local structure around each residue, scores above 90 indicate high confidence, 70–90 represent confident backbone predictions, while scores below 70 suggest low confidence, typically associated with flexible or disordered regions [23,56]. For each sequence, the model with the higher average pLDDT score was selected for subsequent analysis. Prediction reliability was assessed by analyzing the per-residue average pLDDT values and pTM across norovirus genogroups and major genotypes.

### Structure-guided P-domain phylogenies

A joint sequence-structure phylogeny was constructed to better understand evolutionary relationships. First, the *structureto3didescriptor* command from FoldSeek was used to generate 3Di structural descriptors for all P-domain structures. The corresponding AA and 3Di sequences for each structure were extracted and aligned. These aligned sequences were then concatenated into a joint alignment and partitioned into AA and 3Di segments. IQTREE was used to reconstruct the phylogeny from this partitioned alignment under the MFP+MERGE algorithm, which automatically selects the best-fit substitution model and partition scheme while allowing for model merging between partitions. Branch support was evaluated with 1000 UltraFast bootstrap replicates. Based on the best-fit models identified for the AA and 3Di partitions during joint tree inference, separate phylogenetic trees were reconstructed using the AA-only and 3Di-only alignments. To ensure comparability, all other parameters were kept consistent with those used during joint tree construction.

### Structure homology searches

A custom structural database was constructed from all input P-domain structures using the *foldseek createdb* command. Structural comparisons were subsequently conducted using Foldseek software in exhaustive search mode (*--alignment-type 1*). The resulting alignments were processed with the *convertalis* utility to extract the aligned AA sequences, e values, bit score and RMSD values for each pair. RMSD quantifies the average distance between the Cα atoms of superimposed protein structures, calculated as the square root of the mean squared distances between corresponding atoms after optimal structural alignment. Lower RMSD values indicate higher structural similarity. Global RMSD values were extracted to assess overall structural divergence between genogroups or major genotypes. To pinpoint local variations, normalized per-residue RMSD contributions were computed by decomposing the global RMSD, assigning a deviation value to each aligned residue while maintaining consistency with the overall structural alignment.

### Selective pressure analysis

Sequences with 100% identity were first removed using CD-HIT to reduce redundancy. The remaining sequences were translated to AA sequences using EMBOSS transeq, followed by multiple sequence alignment using MAFFT and then back-translated to their corresponding codon-aligned nucleotide alignments using PAL2NAL [57]. Selective pressure was subsequently assessed using three methods implemented in the HyPhy software package: FEL, FUBAR, and MEME. Sites with a p-value < 0.05 in MEME or FEL, or a posterior probability > 0.9 in FUBAR, were considered under significant positive selection.

### Covarying analysis

We performed coevolution analysis of GII.4 VP1 using the Bayesian Graphical Model (BGM) implemented in the HyPhy. The maximum likelihood phylogeny inferred from the same alignment was used as the input tree. BGM was run with 100,000 MCMC steps following a 10,000-step burn-in, with 100 samples collected from the chain. The maximum number of parents per node was set to 1. Site pairs with posterior probability >0.9 and at least two shared substitutions were considered significant evidence of coevolution [58,59]. The resulting coevolving pairs were visualized as an interaction network using R, with node sizes scaled by degree and edge colors representing Cα distances calculated from the predicted GII.4 P-domain structure.

## Supporting information

S1 Fig Distribution of norovirus genogroups and major genotypes. (A) Proportions of genogroups. GII is the predominant genogroup, accounting for over 88% of cases. (B-C) Temporal shifts in the predominant genotypes within GII and GI. Over the past decade, GII.4[P16] and GII.4[P31] have emerged as the most prevalent genotypes. Only the major types are displayed, less common types, such as GII.2[P21], are included in “Others.”
(TIF)

S2 Fig Phylogenetic entanglement tree based on VP1 and RdRp. Inter-genogroup recombination is observed, e.g., GIX and GVIII share VP1 distantly related to GII but harbor GII.P RdRp. Within the predominant GII genogroup, complex recombination patterns are evident, including GII.4 VP1 recombining with multiple P-types (e.g., GII.P4, GII.P31, GII.P16, GII.P12) and GII.17 VP1 with GII.P31 and GII.P17.
(TIF)

S3 Fig Phylogenetic tree of norovirus VP1 AA sequences. The topology shows that each genogroup and the major genotypes each forms a distinct monophyletic clade. Branches are colored according to genogroup assignment.
(TIF)

S4 Fig Structural variability of epitope D region in GII.4 VP1 models. (A) Conformational comparisons among top-confidence models of representative GII.4 variants (New Orleans, Den Haag, and Sydney). (B) Conformational comparisons among five AlphaFold models generated from the same Sydney variant sequence. The high RMSD values observed for epitope D are largely attributable to intrinsic prediction uncertainty in this flexible region.
(TIF)

S1 Table Significant co‑evolving pairs in the GII.4 P‑domain identified by BGM analysis. Listed are all site pairs with posterior probability > 0.9. For each pair, BGM site number (corresponding to the aligned amino acid position in P domain) and the posterior probability of the co‑evolving relationship are provided. Sites located within known antigenic epitopes are indicated (bright green representing Epitope A, purple representing G, orange representing C, light blue representing D, dark green representing E, and dark blue representing H; font in red indicates positively selected sites.). The table also includes the Cα distance (Å) between the two residues, calculated from the AlphaFold2‑predicted structure of the GII.4 Sydney P‑domain.
(DOCX)
