# Supplementary figures and images for: Balancing under constraint: Structural insights into norovirus evolution and antigenic innovation
Source: PLoS Pathog. 2026 Jun 30;22(6):e1014383. doi: 10.1371/journal.ppat.1014383 (PMC13318009; doi:10.1371/journal.ppat.1014383)

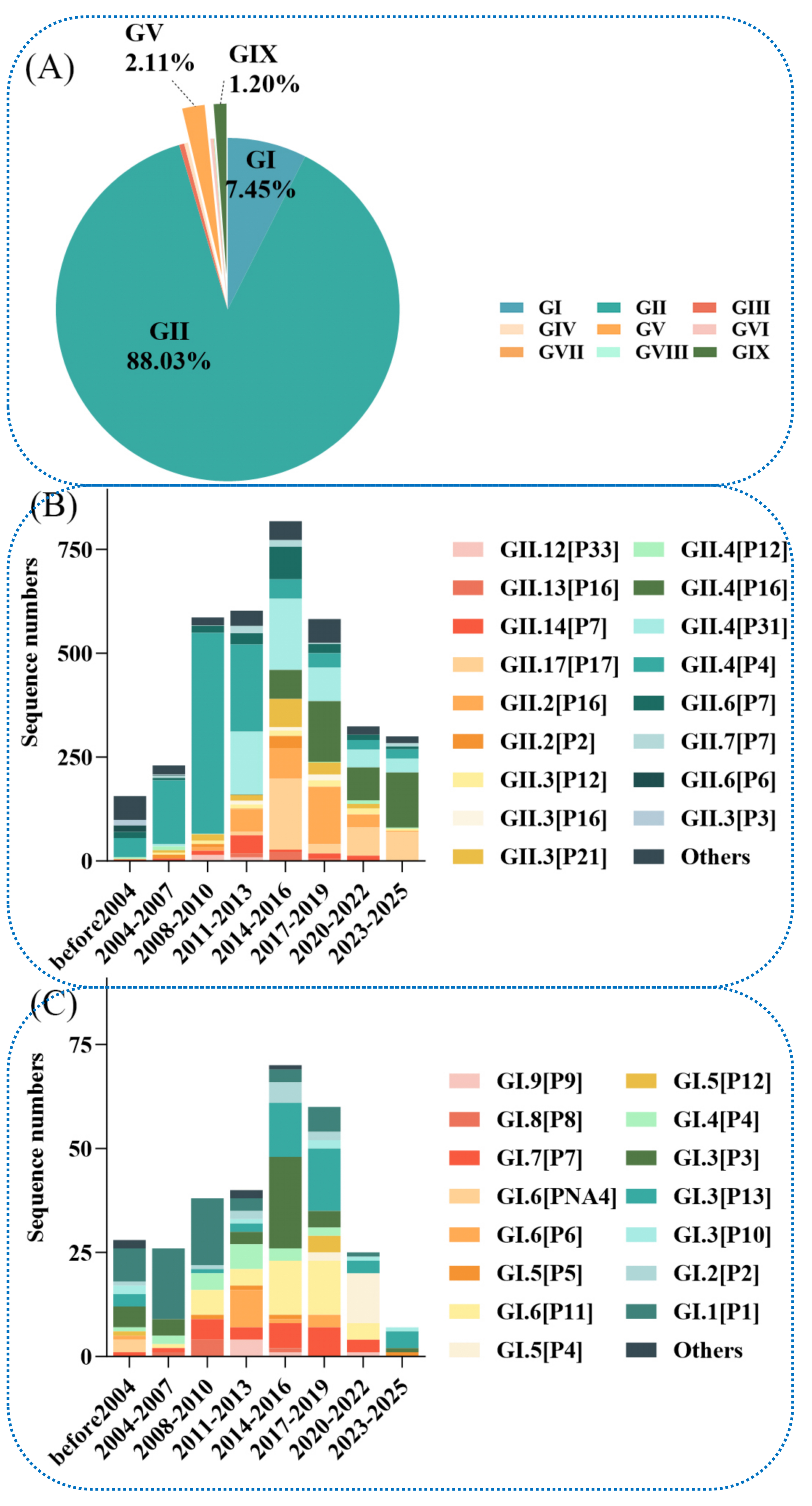

Supplement: S1 Fig — (A) Proportions of genogroups. GII is the predominant genogroup, accounting for over 88% of cases. (B-C) Temporal shifts in the predominant genotypes within GII and GI. Over the past decade, GII.4[P16] and GII.4[P31] have emerged as the most prevalent genotypes. Only the major types are displayed, less common types, such as GII.2[P21], are included in “Others.” (TIF) [file ppat.1014383.s001.tif]

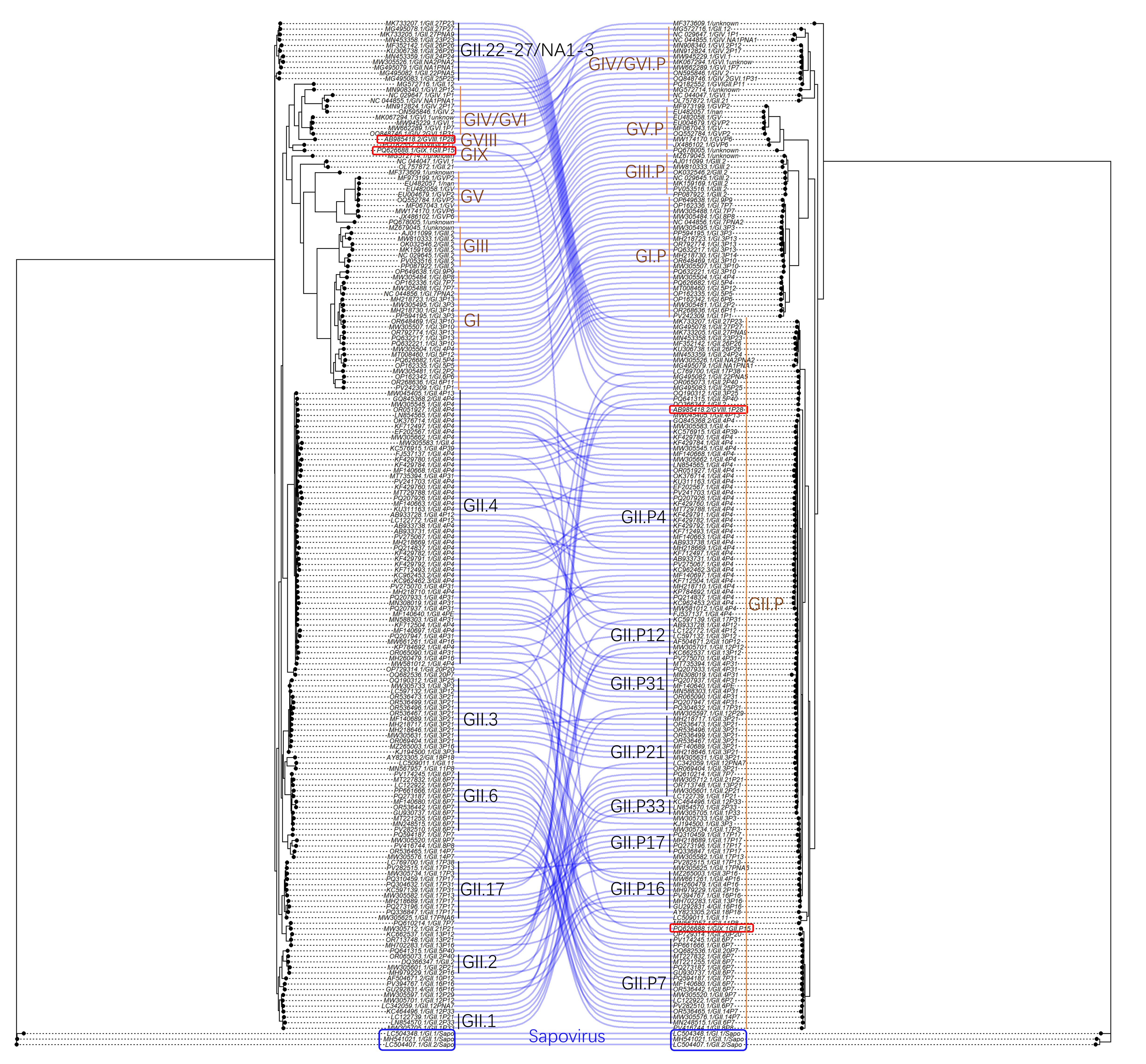

Supplement: S2 Fig — Inter-genogroup recombination is observed, e.g., GIX and GVIII share VP1 distantly related to GII but harbor GII.P RdRp. Within the predominant GII genogroup, complex recombination patterns are evident, including GII.4 VP1 recombining with multiple P-types (e.g., GII.P4, GII.P31, GII.P16, GII.P12) and GII.17 VP1 with GII.P31 and GII.P17. (TIF) [file ppat.1014383.s002.tif]

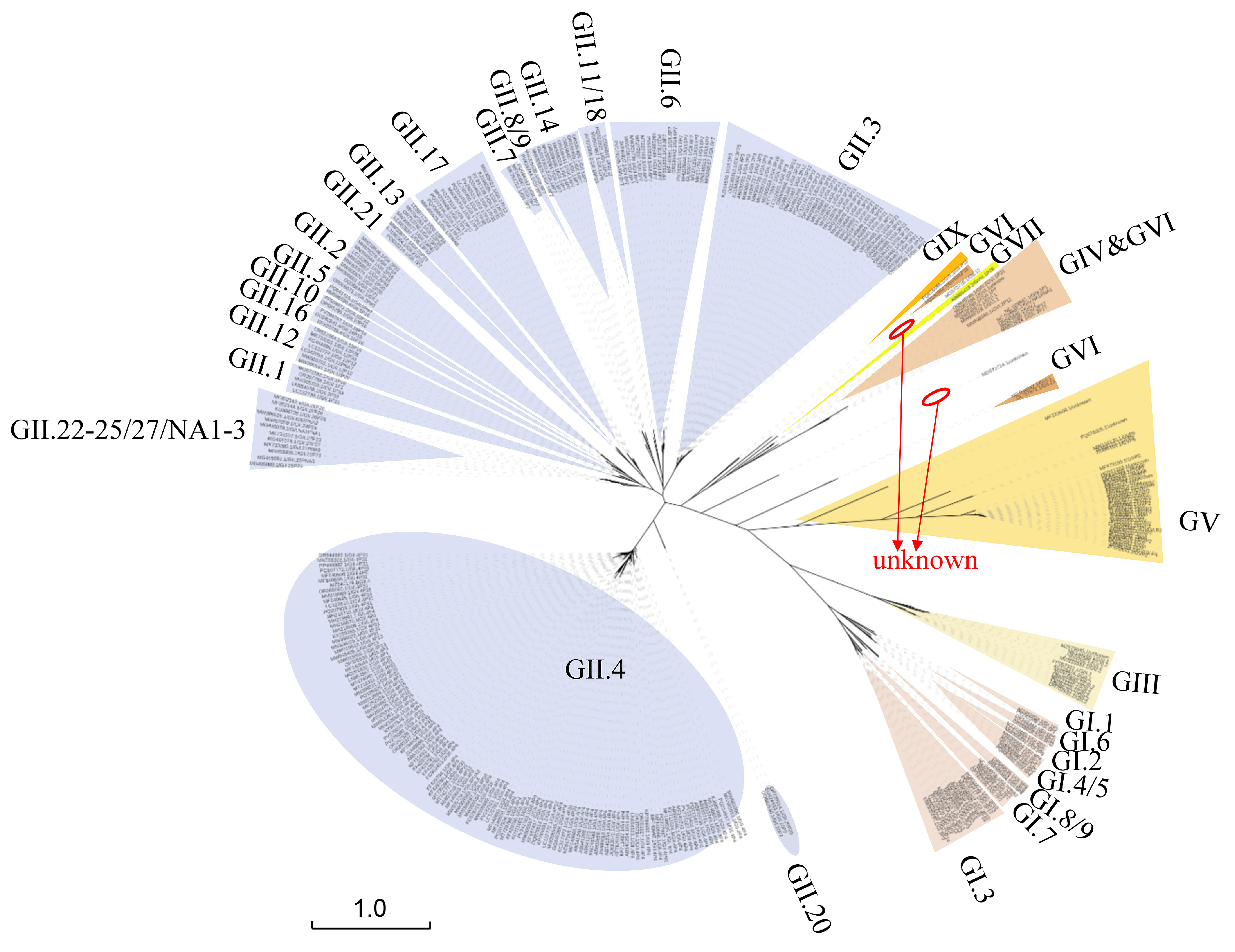

Supplement: S3 Fig — The topology shows that each genogroup and the major genotypes each forms a distinct monophyletic clade. Branches are colored according to genogroup assignment. (TIF) [file ppat.1014383.s003.tif]

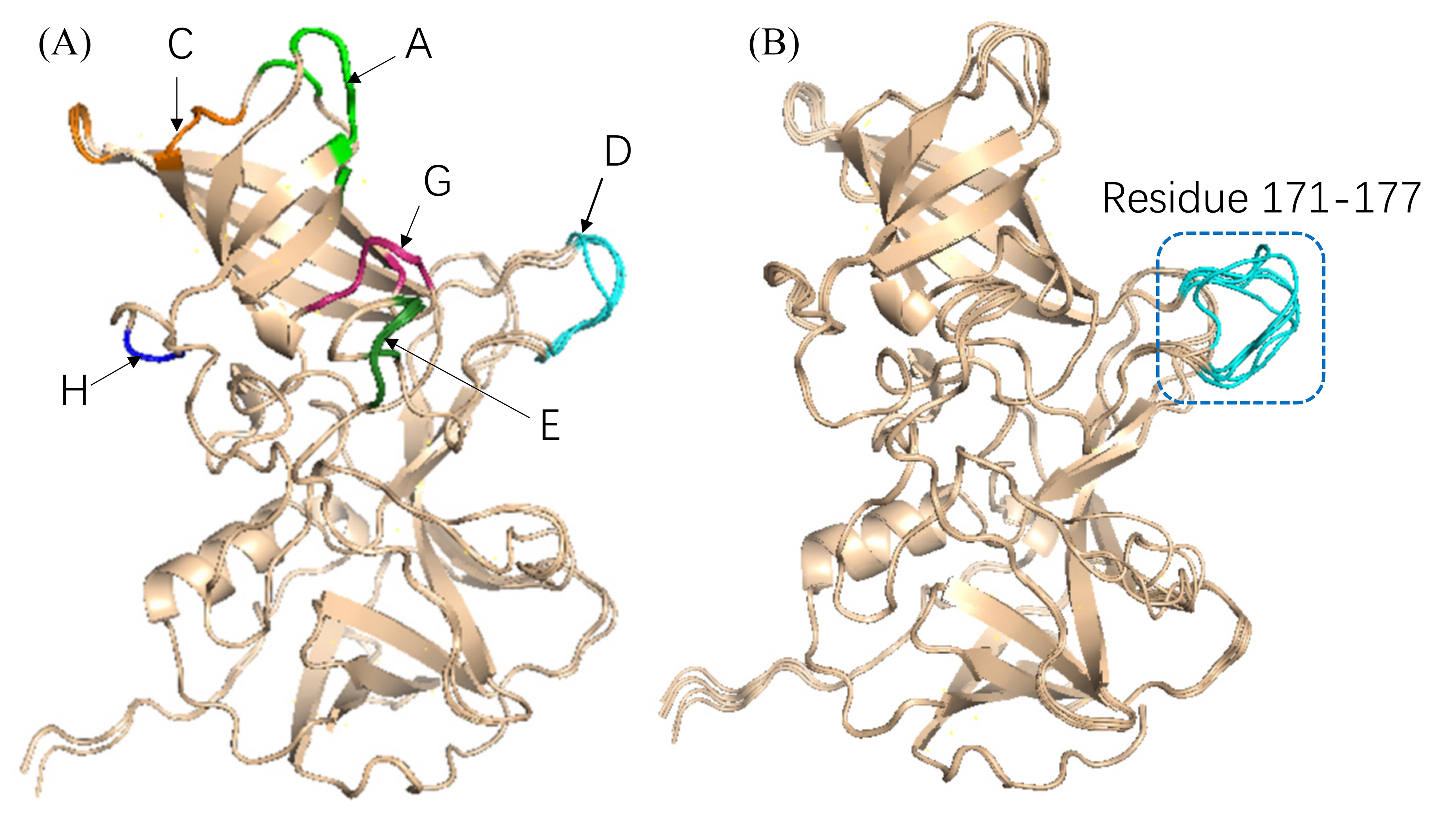

Supplement: S4 Fig — (A) Conformational comparisons among top-confidence models of representative GII.4 variants (New Orleans, Den Haag, and Sydney). (B) Conformational comparisons among five AlphaFold models generated from the same Sydney variant sequence. The high RMSD values observed for epitope D are largely attributable to intrinsic prediction uncertainty in this flexible region. (TIF) [file ppat.1014383.s004.tif]
