# Supplementary material for: Balancing under constraint: Structural insights into norovirus evolution and antigenic innovation
Source: PLoS Pathog. 2026 Jun 30;22(6):e1014383. doi: 10.1371/journal.ppat.1014383 (PMC13318009; doi:10.1371/journal.ppat.1014383)
Supplement: S1 Table — Listed are all site pairs with posterior probability > 0.9. For each pair, BGM site number (corresponding to the aligned amino acid position in P domain) and the posterior probability of the co‑evolving relationship are provided. Sites located within known antigenic epitopes are indicated (bright green representing Epitope A, purple representing G, orange representing C, light blue representing D, dark green representing E, and dark blue representing H; font in red indicates positively selected sites.). The table also includes the Cα distance (Å) between the two residues, calculated from the AlphaFold2‑predicted structure of the GII.4 Sydney P‑domain. (DOCX) [file ppat.1014383.s005.docx]

**S1 table. Significant co‑evolving pairs in the GII.4 P‑domain identified by BGM analysis.**

| **Co-variation pairs** | | **Cα distance (Å)** |  |
| --- | --- | --- | --- |
| **Site 1** | **Site 2** |  | **Posterior** |
| 28 | 35 | 15.1 | 0.996 |
| 28 | 185 | 21.9 | 0.999 |
| 28 | 278 | 15.2 | 0.942 |
| 36 | 112 | 25.7 | 0.999 |
| 36 | 185 | 11.2 | 0.98 |
| 37 | 72 | 41.1 | 0.932 |
| 72 | 76 | 12.1 | 1 |
| 72 | 108 | 23.7 | 0.956 |
| 72 | 159 | 6.3 | 0.973 |
| 73 | 149 | 10.6 | 1 |
| 75 | 158 | 10.5 | 0.994 |
| 77 | 87 | 26.7 | 0.967 |
| 77 | 153 | 5.5 | 0.995 |
| 78 | 153 | 4.8 | 1 |
| 79 | 120 | 22.2 | 0.98 |
| 79 | 147 | 6.7 | 1 |
| 79 | 175 | 27 | 0.952 |
| 79 | 285 | 46.5 | 0.979 |
| 98 | 285 | 38.9 | 0.957 |
| 122 | 177 | 45.1 | 1 |
| 138 | 149 | 11.5 | 0.999 |
| 149 | 153 | 9.7 | 0.974 |
| 157 | 174 | 35.8 | 0.939 |
| 160 | 229 | 34.9 | 0.952 |
| 172 | 182 | 12.2 | 0.968 |
| 172 | 219 | 14.9 | 0.95 |
| 173 | 179 | 8.5 | 0.996 |
| 174 | 194 | 33.1 | 0.948 |
| 178 | 229 | 9.4 | 1 |
| 185 | 223 | 21.2 | 0.979 |
| 219 | 283 | 28.1 | 0.986 |

Notes: The colored fonts indicate epitope sites, with bright green representing Epitope A, purple representing G, orange representing C, light blue representing D, dark green representing E, and dark blue representing H; underlined numbers indicate positively selected sites.
